# Perceived changes of specific attitudes, perceptions and behaviors during the Corona pandemic and their relation to wellbeing

**DOI:** 10.1186/s12955-020-01623-6

**Published:** 2020-11-30

**Authors:** Arndt Büssing, Daniela Rodrigues Recchia, Rudolf Hein, Thomas Dienberg

**Affiliations:** 1 Professorship Quality of Life, Spirituality and Coping, Faculty of Health, Witten/Herdecke University, 58313 Herdecke, Germany; 2 IUNCTUS - Competence Center for Christian Spirituality, Philosophical-Theological Academy, 48149 Münster, Germany; 3 Chair of Research Methods and Statistics in Psychology, Faculty of Health, Witten/Herdecke University, 58448 Witten, Germany

**Keywords:** Changes of perceptions, Corona pandemic, Wellbeing, Life satisfaction, Change of attitudes, Spirituality, Awe, Gratitude

## Abstract

**Background:**

During the COVID-19 pandemic, most people had to cope with the restrictions of the lockdown, leaving them to their fears, insecurity and isolation. On the other hand, due to the unexpected ‘extra time’ there was room for new experiences and for personal reflections on what is essential in life, to perceive nature and relations more consciously etc. We, therefore, intended to analyze perceived changes of attitudes and behaviors during the time of lockdown, and whether these perceptions would contribute to personal wellbeing during the pandemic.

**Methods:**

An anonym cross-sectional online survey was performed for data collection, using standardized questionnaires, i.e., the WHO-Five Well-being Index (WHO-5), Brief Multidimensional Life Satisfaction Scale (BMLSS), Awe/Gratitude scale (GrAw-7), and the newly developed *Perceived Changes Questionnaire* (PCQ).

**Results:**

Within the number of respondents (n = 1277), women were predominating (67.5%). Participants’ mean age was 50.9 ± 14.9 years. Exploratory factor analyses showed that the 24-item *Perceived Changes Questionnaire* differentiated five factors that would account for 61% of variance: (1) *Nature/Silence/Contemplation* (Cronbach’s alpha = .87), (2) *Spirituality* (Cronbach’s alpha = .83), (3) *Relationships* (Cronbach’s alpha = .80), (4) *Reflection on life* (Cronbach’s alpha = .74), (5) *Digital media usage* (Cronbach’s alpha = .74). Strongest changes were observed for *Relationships* and *Nature/Silence/Contemplation*. Perceived changes were stronger among older persons, among persons with higher wellbeing, and among those who relied on their faith as a resource. These changes were predicted best by a person’s perception of wondering awe in distinct situations with subsequent feelings of gratitude. Stepwise regression analyzes revealed that participants’ wellbeing was explained best by low perceived burden and high life satisfaction (R^2^ = .46). Awe/gratitude, perceived changes in terms of *Nature/Silence/Contemplation* and low *Reflections of live* are further variables that would predict a person’s wellbeing among the COVID-19 pandemic.

**Conclusions:**

During the Corona pandemic, people tried to find ways to adapt to the outcomes of the restrictions. The perceived changes of attitudes and behaviors can be interpreted in terms of a reappraisal strategy. These can be measured with the extended version of the PCQ which was found to have good quality indices and a plausible factor structure. The reported changes contribute to persons’ wellbeing only to some extend, indicating that they represent an independent quality of relevance in peoples’ life.

## Introduction

Like in almost every country in the world, the severity of the COVID-19 pandemic brought about in Germany a complete social and economic lockdown in spring 2020. The public health system has focused on diagnosis, quarantine, and supportive treatment possibilities for infected patients [1, 2]. Managing of people at risk was seen as a challenging task by health professional, because there is currently no cure or reliable treatments established [3, 4]. This insecurity resulted in fears and worries among the general population, too, because the only preventive option seems to be personal hygiene and social distancing. Several might thus experience phases of loneliness, depression or ‘defeat stress’ which is characterized by an inability to “cope with adversity” [5]. Most persons in Germany complied with the individual and social restrictions, implemented by the government, generally staying in their homes. As a consequence, especially the elderly people (and many others) felt isolated from their friends and relatives [6]. Others missed the collaborative networks at their distant workplace and had to deal with so much (boring) ‘extra time’. Some experienced a degree of fear getting into contact with potentially infected persons [4, 7]. So, they avoided direct contact and allowed themselves to go to the grocery and pharmacies only (which actually was in some countries the strictest form of lockdown). Persons, suffering from chronic diseases and also those with acute symptoms of illness refrained from visiting hospitals, were very hesitant to visit their medical practitioners simply because they were afraid to risk a viral infection [4, 7].

Persons in lockdown had to cope with their own fears and insecurity and the outcomes of being isolated on the one hand (which can be regarded as stressors or burden), while on the other hand, they also made new experiences, as they had a lot of ‘extra time’ for personal reflections about what is seen as essential in their life, to perceive nature and their relations more consciously etc. (which can be seen as putative resources to cope). We, therefore, intended to analyze the perceived changes of attitudes and behaviors during the time of lockdown. Flash interviews among tumor patients from Germany have documented the restrictions in daily life and loneliness as the main burdens during the crisis [4]. With those taking part in the survey, faith proved to be a major factor of stability and stronghold.

What about perceived changes in a non-diseases group of persons? Apart from concerns of loneliness and feeling restricted, many used the ‘extra time’ of the lockdown to spend more time outdoors, to perceive nature more intensely, to spend more time with their partner and their children—and generally to have more time for themselves. This ‘extra time’ could be used as a chance to reflect on those things which may give meaning in life, to reflect on what is essential in life, maybe also as a hint to change important aspects of life, to be more aware of nature and of people in the neighborhood, and to deal more consciously (‘mindfully’) with them. Further, some persons during the Corona pandemic have experienced that these restricted times allowed them to focus more on their own spiritual resources (meditation/prayer), and, thus, some have enjoyed the ‘silence’, while others feared this ‘silence’ because they became aware of their loneliness and insecurity. These experiences can be related to the concepts of ‘spiritual transformation’ [8, 9] or ‘posttraumatic growth’ [10, 11]. Spiritual transformation was described also in HIV infected persons by Kremer and Ironson [12], with more intense spiritual practices, meaning finding, self-perception and self-knowledge. With HIV-infected persons, spiritual transformation related to better wellbeing, less distress and better coping [13]. Posttraumatic growth in cardiac surgery survivors was predicted by religious coping and partner support [10]; interestingly, perceived spiritual support was a mediator of these effects.

During the Corona pandemic González-Sanguino et al. [14] performed an online survey among people from Spain and found that 18.7% were depressed, 21.6% perceived anxiety and 15.8% had PTSD symptoms. In their study, spiritual wellbeing was the best protector for symptomatology, and loneliness for depression, anxiety and PTSD. In a study among tumor patients from Germany it was found that the topics meaning in life, having (religious) trust, stable relationships, mindful encounter with nature, and times of reflection were important to them to cope with the restrictions during the Corona pandemic [7]. In that study, a short version of the Perceived Changes Questionnaire was used; an extended version of this assessment instrument will be used in this study, that aimed to analyze main factors of spiritual and personal coping strategies during the Corona pandemic in terms of more conscious awareness, religious and personal (social) bonding, particularly in the light of perceived changes of attitudes and behavior linked to relationships, awareness of nature and quietness, interest in spiritual issues, or feelings of worries and isolation. We also asked whether these perceptions would contribute to personal wellbeing during the pandemic.

Despite the fact that this is an explorative analysis of distinct perceptions shaped during the Corona crisis, we had several assumptions relevant for the selection of variables. Research indicates that personal faith can be a relevant resource to cope with difficult times [15–17], and thus, we assume that even within a secular society several may rely on their faith as a strong hold in difficult times to cope, and that this will increase their awareness for the Sacred in their life and conscious encounter also with the world around. Similarly, those who are able to stop and be aware of the beauty around and who are emotionally or ‘spiritually’ touched by specific situations (in terms of wondering awe and subsequent feelings of gratitude) [18, 19], perceive more positive changes in their attitudes and behaviors. Thus, male and female religious living in monastic structures who are (due to their specific life style) trained to be more aware of the Sacred in their life and are more active in spiritual practices and rituals, might be more able to change their attitudes and behaviors compared to persons with other life styles. This group was chosen as a contrasting group to persons from different other professions (i.e., administration, economy, education, health, and other). Thus, we assume that perceived burden due to the COVID-19 pandemic (“Stressors”) may be the *cause* to perceive life concerns differently, while specific attitudes (i.e., having faith as a resource and being able to perceive feeling of wondering awe and gratefulness on the one hand, and frequency of meditation and praying as indicators of a person’s spiritual practice) would be positively associated (“Resource”) as positive influencing variables.

## Material and methods

### Recruitment of patients

Participants were recruited within 4 weeks via snowball sampling in different networks in Germany, i.e., university students and staff, research collaborators, religious orders and church communities, Rotary Club members, Facebook sites, etc. (from June 9 to June 21, 2020). As well, all were invited to spread the information about this survey in their personal networks. In Germany, the lockdown started at March 16; during end of May 2020 several restrictions were relaxed step by step.

Participants were assured confidentially and were informed about the purpose of the study and data protection information at the starting page of the online survey. By filling in the anonymous questionnaire, interested persons consented to participate. Neither concrete identifying personal details nor IP addresses were recorded to guarantee anonymity.

### Measures

#### Perception of changes

The Corona pandemic and related social and individual restrictions may have changed specific attitudes, perceptions and behaviors. To assess which changes due to the Corona pandemic were observed, we used an extended version of the previously tested 12-item short version of the Perception of Change Questionnaire (PCQ) [7]. The 32 statements of this extended version cover the following topics: more intense relations, mindful perception of nature, times of quietness, spirituality, connectedness/loneliness, meaning in life, hope and fear. These refer to the experiences communicated by various persons during the start of the pandemic. And they are related to the concept of ‘spiritual transformation’ [8, 9] and ‘posttraumatic growth’ [11]. The respective items were introduced by the phrase “Due to the current situation…”, which referred to the Corona pandemic. Agreement or disagreement was scored on a 5-point scale (0—does not apply at all; 1—does not truly apply; 2—neither yes nor no; 3—applies quite a bit; 4—applies very much). Items phrasings, factorial structure and internal consistency coefficients are depicted in Table 2.

#### Well-being Index

To assess participants’ well-being, we used the WHO-Five Well-being Index (WHO-5). This short scale avoids symptom-related or negative phrasings and measures well-being instead of absence of distress [20]. Representative items are “I have felt cheerful and in good spirits” or “My daily life has been filled with things that interest me”. Respondents assess how often they had the respective feelings within the last two weeks, ranging from at no time (0) to all of the times (5). Here we report the sum scores ranging from 0 to 25. Scores < 13 would indicate reduced wellbeing or even depressive states. Using the alternative WHO-5 sum scores referred to a 100% level [0–100], which is also used in literature, scores < 50 are indicative for reduced wellbeing and scores < 28 for clinical depression [21]. Using these WHO-5 sum scores [0–100], persons with scores < 13 would have a WHO5 sum score of 33.9 ± 11.7 in this sample, while persons with moderate wellbeing (scores 13–16) would have a sum score of 65.6 ± 6.9, and those with high wellbeing (scores > 18) would have a WHO-5 sum score of 82.4 ± 6.5.

#### Life satisfaction

Life satisfaction was measured using the Brief Multidimensional Life Satisfaction Scale (BMLSS) [22]. The items of the BMLSS address intrinsic (oneself, life in general), social (friendships, family life), external (work situation, where one live) and prospective dimensions (financial situation, future prospects) of life satisfaction as a multifaceted construct. The internal consistency of the instrument was found to be good in the validation study (Cronbach’s alpha = 0.87). In this study, the 10-item version was employed that includes satisfaction with the health situation and abilities to deal with daily life concerns (BMLSS-10). We added two further items addressing satisfaction with support by friends and cohesion among friends.

#### Perception of burden

Perceived restrictions of daily life, of being under pressure/stressed, anxiety/insecurity, loneliness/social isolation and restrictions of financial-economic situation due to corona pandemic were measured with five numeric rating scales (NRS), ranging from 0 (not at all) to 100 (very strong) as described [7]. These five variables can be combined to a factor termed “Stressors” with good internal consistency in this sample (Cronbach’s alpha = 0.801). This “Stressor” scale is strongly related to reduced wellbeing (WHO-5: r = − 0.59) and reduced life satisfaction (BMLSS-10: r = − 0.53) as related constructs in this sample, but marginally only with awe/gratitude (GrAw-7: r = − 0.10) as an unrelated construct.

#### Indicators of spirituality

Perceptions of wondering awe and subsequent gratitude is a perceptive aspect of secular spirituality which is relevant also to less or non-religious persons [18]. To address times of pausing for ‘wonder’ in specific situations and stations (mainly in the nature), we measured perceived awe and subsequent feelings of gratitude as a perceptive aspect of spirituality with the 7-item Awe/Gratitude scale (GrAw-7) [18]. This scale has good psychometric properties (Cronbach’s alpha = 0.82) and uses items such as “I stop and then think of so many things for which I'm really grateful”, “I stop and am captivated by the beauty of nature”, “I pause and stay spellbound at the moment” and “In certain places, I become very quiet and devout”. Thus, awe/gratitude operationalized in this way is a matter of an emotional reaction towards an immediate and ‘captive’ experience. All items were scored on a 4-point scale (0—never; 1—seldom; 2—often; 3—regularly), referred to a 100-point scale.

To measure also a more specific forms of religiosity, we added item A37 from the Reliance on God’s Help scale [23], which asks whether faith is a strong hold in difficult times. Agreement or disagreement was scored on a 3-point scale (0—disagreement; 2—indifference; 3—agreement). This item was used as a differentiating variable to assess intrinsic religiosity in terms of an attitude.

The frequency of spiritual/religious practices such as meditation or praying was assessed with a 4-grade scale ranging from never, to at least once per month, at least once per week, and at least once per day as described [7].

#### Corona pandemic irritations

Several persons reported that they were “Irritated or unsettled by different statements about the danger and the course of the corona infection in the public media” [4, 7]. Agreement to this statement was scored from not at all, a little, somewhat to very much.

#### Health behaviors

Health behaviors such as Alcohol consumption, usage of relaxing (“mood lifting”) drugs, physical activity/sporting, and walking outside in the nature were measured with a 4-grade scale (never, at least once per month, at least once per week, at least once per day) as described [7].

### Statistical analysis

Descriptive statistics, analyses of variance (ANOVA), internal consistency (Cronbach’s coefficient α) and factor analyses (principal component analysis using Varimax rotation with Kaiser’s normalization) as well as first order correlation (Spearman rho) and stepwise regression analyses were computed with SPSS 23.0. Moderation analysis was used to investigate the possible influence of a variable (gender) on the effect of one predictor (awe/gratitude) when regressing on the response variable (Perceived Changes). This is a strategical approach to estimate the conditional effects of a moderator. The moderation analysis was performed with R 4.0.3.

Given the exploratory character of this study, significance level was set at *p* < 0.01. With respect to classifying the strength of the observed correlations, we considered r > 0.5 as a strong correlation, an r between 0.3 and 0.5 as a moderate correlation, an r between 0.2 and 0.3 as a weak correlation, and r < 0.2 as negligible or no correlation.

## Results

### Description of the sample

The online survey was accessed by 1509 persons, and 162 did not start the survey (10.7%). Among the 1374 starting persons, 97 provided only basic sociodemographic data, but did not respond to the subsequent items; these were regarded as non-responders (7.1% of the starters). These non-responders did not significantly differ from the responders with respect to gender, age, lack of religious affiliations or COVID-19 infection testing (data not shown).

Within the responders (n = 1277), women were predominating (67.5%) (Table 1). Participants’ mean age was 50.9 ± 14.9 years. Their professions are divers, ranging from administration, economy, education, medicine, church and other (i.e. coaching, psychology, kindergarden teacher, yoga teacher, journalism, culture, social work, police, agriculture, service, and retired persons). A majority had a Christian affiliation (76%), and 19% none. Nevertheless, only for 51% their faith was a strong hold in difficult times.

**Table 1:** Sociodemographic data of participants (N = 1277)

	n	% of responders	Mean ± SD	Range
*Gender*				
Women	859	67.5		
men	414	32.5		
Age (years)	1261		50.9 ± 14.9	15–92
*Living conditions*				
Family household	534	41.8		
Shared house	131	10.3		
Single	252	19.7		
Retirement home	1	0.1		
Monastery/community	162	12.7		
*Profession*				
Administration	181	14.2		
Economy	134	10.5		
Education	178	13.0		
Medicine/health	234	17.0		
Church	314	22.9		
Other	391	28.5		
*Corona tested*				
Positively tested	10	0.8		
Negatively tested	103	8.1		
No testing	1164	91.2		
*Irritated or unsettled by different statements about the danger and the course of the corona infection in the public media*	1277		1.4 ± 0.9	0–3
Not at all	220	17.2		
A little	493	38.6		
somewhat	389	30.5		
very much	175	13.7		
*Religious affiliation*				
Catholics	740	57.9		
Protestant	235	18.3		
Other	72	5.7		
None	241	18.9		
*Faith as strong hold in difficult times*				
Disagreement	271	21.5		
Undecided	344	27.2		
Agreement	648	51.3		
Awe/gratitude (GrAw-7)	1267		66.8 ± 17.9	0–100
*Meditation*			Meditation was practices quite often 30%	
Never	407	33.9		
At least once per month	176	14.6		
At least once per week	257	21.4		
At least once per day	362	30.1		
*Praying*			Praying was often used quite often 49%	
Never	321	26.8		
At least once per month	121	10.1		
At least once per week	175	14.6		
At least once per day	581	48.5		
*Mood lifting drugs*			Mood lifting drugs were rarely used 4%	
Never	1129	95.4		
At least once per month	10	0.8		
At least once per week	10	0.8		
At least once per day	34	2.9		
*Alcohol consumption*			Daily alcohol consumption was rather rare 8%	
Never	312	25.8		
At least once per month	355	29.4		
At least once per week	442	36.6		
At least once per day	99	8.2		
*Physical activity/sporting*				
Never	170	14.2		
At least once per month	162	13.5		
At least once per week	633	52.8		
At least once per day	234	19.5		
*Walking outside in nature*				
Never	36	2.8		
At least once per month	169	13.2		
At least once per week	631	52.2		
At least once per day	373	30.9		
*Wellbeing and burden*				
Wellbeing (WHO-5 100)	1268		60.6 ± 20.8	0–100
Wellbeing (WHO-5 sum)	1268		15.1 ± 5.2	0–25
WHO-5 sum scores < 13	376	29.7		
WHO-5 sum scores 13–18	501	39.5		
WHO-5 sum scores > 18	391	30.8		
Life satisfaction (BMLSS-10)	1266		68.1 ± 15.1	0–100
Daily life restrictions (NRS)	1265		47.3 ± 26.2	0–100
Under pressure/stress (NRS)	1231		35.0 ± 29.5	0–100
Anxiety/insecurity (NRS)	1258		22.8 ± 23.8	0–100
Loneliness/social isolation (NRS)	1240		23.9 ± 27.4	0–100
Financial-economic situation (NRS)	1257		18.6 ± 28.0	0–100

Several were irritated or unsettled by different statements about the danger and the course of the corona infection in the public media (44% somewhat to very much). A COVID-19 infection was found in 0.8% (10 persons), 8% were negatively tested, most not at all (91%) (Table 1). Within the non-completers, five persons were COVID-19 infected, 9 persons negatively tested and 78 not yet tested.

### Wellbeing and health behavior in the sample

With respect to the generally accepted standard that WHO-5 scores < 13 may indicate low wellbeing or even depressive states, in our sample 30% would have had low wellbeing (WHO-5 score < 13); 40% moderate wellbeing (WHO-5 scores between 15 and 18) and 31% high wellbeing (WHO-5 scores > 18). General life satisfaction was in the upper third, while perceived daily life restrictions were in a moderate range. Feelings of being under pressure/stress, anxiety/insecurity, loneliness/social isolation and restrictions by financial-economic situation were in the lower range, indicating only weak burden (Table 1).

With respect to respondents’ health behavior, Alcohol consumption was found 37% at least once per week and 8% at a daily level, while mood lifting drugs were rarely used (3% at least once per day). Physical activity/sporting was found by 53% at least once per week and by 20% at a daily level. Walking in nature was found by 52% at least once per week and by 31% at a daily level. Further, meditation was practiced by 21% at least once per week and by 30% at a daily level, while praying was practiced by 15% at least once per week and by 49% at a daily level (Table 1).

### Perception of changes of attitudes and behaviors

To better summarize and calculate patients’ perceived changes in attitudes and behavior, we intended to combine these as factors and thus performed reliability and factor analyses of the 32 intended items. A Kaiser–Mayer–Olkin value of 0.89 (as a measure for the degree of common variance) indicated that the item pool is suited for principal component factor analysis.

During this process some items were eliminated: Three items referring to the intended topic of isolation were eliminated due to a weak corrected item to scale correlation (c15 I feel cut off from life; c16 I feel restricted in my freedom; c17 I lack social contacts); however, these three had an acceptable internal consistency (Cronbach’s alpha = 0.78) and were used as an addition scale to address perceived “Restrictions”. Also, three items referring to perceived loss had to be eliminated because of a too weak reliability (c27 I find that our society is falling apart more and more; c28 I rather fear for the future; c30 I lost my belief); their internal consistency as a putative factor was not satisfactory (Cronbach’s alpha = 0.53). Exploratory factor analysis pointed to six factors with eigenvalues < 1, among them a 2-item factor which refers to the two “hope” items c25 (“I have the hope that we (‘afterwards’) as global mankind will pay more attention to each other and stick together”) and c26 (“I would like to work to ensure that the world becomes fairer in the future”); however, their internal consistency was not satisfactory (Cronbach’s alpha = 0.57) and both items were eliminated from the item pool. The remaining 24 items had a very good internal consistency (Cronbach’s alpha = 0.91) and differentiated in five factors that would account for 61% of variance (Table 2):

1. *Nature*/*Silence*/*Contemplation* (7 items; Cronbach’s alpha = 0.868), combines the topics experience of silence and mindful perceptions, i.e., taking time for silence, enjoying times of silence, going outdoors more often and perceive nature more intensely, and subsequently more time to reflect what is really important in life and to deal more consciously with own concerns, and being more relaxed than before
2. *Spirituality* (5 items; Cronbach’s alpha = 0.827), refers to interest in spiritual issues, confidence in a higher support, praying, and attending digital worship/service offers
3. *Relationships* (6 items; Cronbach’s alpha = 0.800), includes all items addressing connectedness in its various forms, i.e., taking more time for my family and friends, perceiving the relationship with my partner, family and friends more intensely, feeling closer to the people in my household, importance of relationships to feel safe and at home, and the intention to be more friendly with others
4. *Reflection on life* (3 items; Cronbach’s alpha = 0.744), combines two meaning items (concerned about meaning and purpose of life and concerned about the lifetime one has) and one isolation/loneliness item (more intensive perception of loneliness)
5. *Digital media usage* (3 items; Cronbach’s alpha = 0.742), refers to digital forms of connectedness (i.e., which allow to share in the world, to connected to friends, and to be inspired by specific websites).

**Table 2:** Factor and reliability analyses (24 items)

Intended topic	Items	Mean value	SD	Difficulty index (2.33/4 = 0.58)	Corrected item—scale correlation	Cronbach’s alpha If item deleted (alpha = .908)	Factor loading				
							1	2	3	4	5
	Eigenvalue						7.6	2.3	1.7	1.4	1.3
	Cronbach’s alpha						.868	.827	.800	.744	.742
	*Factor 1: Nature/Silence/Contemplation*										
Silence	c12 I consciously take more time for silence	2.15	1.17	0.54	.671	.901	.724	.382			
Mindfulness	c11 I perceive nature more intensely	2.63	1.10	0.66	.632	.902	.710				
Mindfulness	c10 I go outdoors much more often	2.61	1.12	0.65	.497	.904	.678				
Mindfulness	c8 I come to deal more with myself again	2.44	1.06	0.61	.547	.903	.663				
Silence	c13 I enjoy quiet times of reflection	2.29	1.21	0.57	.663	.901	.649	.439			
Mindfulness	c9 I pay more attention to what's really important in life	2.72	1.00	0.68	.648	.902	.615		.362		
Mindfulness	c7 I'm more relaxed than before	2.00	1.04	0.50	.344	.907	.579			− .302	
	*Factor 2: Spirituality*										
Spirituality	c32 I have confidence in a higher power that supports me	2.69	1.39	0.67	.514	.904		.791			
Spirituality	c31 I deal more with spiritual/religious questions	1.79	1.22	0.45	.622	.902		.755			
Spirituality	c20 I'm more interested in spiritual/religious issues	1.77	1.19	0.44	.649	.901		.733			
Spirituality	c29 I pray/meditate more than before	1.70	1.23	0.43	.612	.902	.322	.692			
Spirituality	c22 I took advantage of digital worship services	1.54	1.58	0.39	.340	.909		.643			
	*Factor 3: Relationships*										
Connectedness	c1 I perceive the relationship with my partner/family more intensely	2.72	1.00	0.68	.431	.906			.775		
Connectedness	c4 I feel closer to the people in my household	2.69	1.07	0.67	.417	.906			.748		
Connectedness	c3 I take more time for my family/friends	2.51	1.03	0.63	.508	.904			.694		
Connectedness	c5 relationships have become important to me in which I can feel safe and at home	2.89	0.98	0.72	.571	.903			.654		
Connectedness	c2 I perceive the relationships with my friends more intensely	2.34	1.04	0.59	.443	.905			.560		
Mindfulness	c6 I try to be more friendly with others	2.61	0.93		.526	.904	.326		.508		
	*Factor 4: Reflection on life*										
Meaning	c24 I'm more concerned about the meaning and purpose of my life	2.29	1.21	0.57						.793	
Meaning	c23 I'm more concerned about the lifetime that I have	2.23	1.24	0.56						.784	
Isolation	c14 I perceive times of loneliness more intensely	2.05	1.26		.413	.906	.322			.529	
	*Factor 5: Digital media usage*										
Connectedness	c19 I use many internet offers that let me share in the world	2.29	1.20	0.57	.372	.907					.881
Connectedness	c18 I am connected to friends via digital media	2.76	1.05	0.69	.345	.907					.778
Spirituality	c21 I use more and more websites that inspire and stimulate me	1.91	1.20	0.48	.509	.904		.405			.637
	*Deleted items*										
Isolation	c15 I feel cut off from life	1.15	1.25								
Isolation	c16 I feel restricted in my freedom	1.82	1.33								
Isolation	c17 I lack social contacts	2.13	1.34								
Hope	c25 I have the hope that we (‘afterwards’) as global mankind will pay more attention to each other and stick together	2.47	1.21								
Hope	c26 I would like to start working to ensure that the world becomes fairer in the future	2.73	0.94								
Loss	c27 I find that our society is falling apart more and more	2.54	1.01								
Loss	c28 I rather fear for the future	1.78	1.13								
Loss	c30 I lost my belief	0.50	0.86								

Extraction method: principle component analysis (Varimax rotation with Kaiser normalization). Rotation is converged in 6 iterations; the 6 factors explain 61% of variance

The difficulty index of these 24 items was (mean score 2.33/4 =) 0.58; all were in the acceptable range of 0.2 and 0.8 (Table 2).

### Perceptions of change within the sample

The most frequently perceived changes were more intense *Relationships* and *Perception of Nature*/*Silence*/*Contemplation*, followed by *Reflection on life* and *Digital media usage*, and *Spirituality,* while *Perceived Restrictions* scored lowest (Table 3).

**Table 3:** Strength of perceived changes within different sub-groups

		Perceived changes					
		Nature/Silence/Contemplation	Spirituality	Relationships	Reflection on life	Digital media usage	Restrictions
		60.28	47.66	65.74	55.06	57.99	42.54
		20.50	25.95	18.26	25.42	23.57	27.40
*Gender*							
Female	Mean	62.15	48.92	66.65	57.07	58.32	42.45
	SD	20.79	26.07	18.29	25.27	24.38	27.58
Male	Mean	56.55	45.20	63.86	50.93	57.30	42.57
	SD	19.41	25.57	18.10	25.28	21.93	26.86
F value		**20.26**	5.51	6.23	**15.71**	0.49	0.01
*p* value		**< .0001**	.019	.013	**< .0001**	n.s	n.s
*Age cohorts*							
< 30 years	Mean	54.27	30.35	63.70	52.59	57.50	51.99
	SD	19.37	24.50	16.79	25.00	23.64	27.65
30–40 years	Mean	52.29	35.93	61.60	45.20	57.82	43.29
	SD	20.97	27.10	19.20	26.60	21.69	27.51
41–50 years	Mean	57.98	46.89	64.13	51.04	57.41	44.26
	SD	21.09	25.27	18.02	26.15	24.24	28.81
51–60 years	Mean	61.92	51.71	66.97	55.65	57.70	40.64
	SD	20.19	22.60	18.34	24.47	24.02	25.68
61–70 years	Mean	63.70	51.89	66.26	58.43	57.93	40.36
	SD	18.85	22.92	17.64	23.79	22.18	28.34
> 70 years	Mean	70.54	65.14	72.28	70.54	60.82	33.96
	SD	17.80	26.43	18.62	22.20	26.09	23.78
F value		**14.93**	**35.84**	**5.18**	**14.51**	0.33	**6.51**
*p* value		**< .0001**	**< .0001**	**< .0001**	**< .0001**	n.s	**< .0001**
*Religious*							
Living in Monastery	Mean	65.52	63.37	66.86	60.68	61.49	35.62
	SD	21.09	21.41	18.63	25.30	25.84	25.89
All other	Mean	59.51	45.36	65.58	54.25	57.50	43.52
	SD	20.31	25.77	18.21	25.34	23.20	27.48
F value		**11.73**	**68.74**	0.65	**8.60**	3.76	**11.07**
*p* value		**.001**	**< .0001**	n.s	**.003**	.053	**.001**
*Faith as a strong hold*							
Does not apply	Mean	52.72	19.50	60.02	46.94	54.22	43.82
	SD	22.37	22.88	20.20	28.18	24.03	28.44
Partly	Mean	58.30	44.05	65.01	55.68	56.95	47.32
	SD	18.76	19.22	16.00	23.82	22.11	26.86
Applies	Mean	64.45	60.91	68.62	58.24	60.28	39.81
	SD	19.63	19.77	17.79	24.31	24.04	26.84
F value		**33.13**	**377.52**	**21.22**	**18.40**	**6.46**	**8.43**
*p* value		**< .0001**	**< .0001**	**< .0001**	**< .0001**	**.002**	**< .0001**
*Wellbeing (WHO-5)*							
Scores < 13	Mean	55.42	45.05	63.26	60.41	56.80	57.50
	SD	19.51	24.72	16.68	24.56	23.09	27.04
Scores 13–18	Mean	59.16	46.36	65.56	53.08	58.08	41.14
	SD	19.70	25.30	18.32	25.22	22.69	25.12
Scores > 18	Mean	66.08	51.67	68.38	52.66	59.06	30.06
	SD	20.97	27.38	19.16	25.76	25.09	23.64
F value		**26.90**	**6.96**	**7.32**	**11.19**	0.84	**108.68**
*p* value		**< .0001**	**.001**	**.001**	**< .0001**	n.s	**< .0001**

Differences p < 0.01 are highlighted (bold)

Women scored significantly higher on *Nature*/*Silence*/*Contemplation* and *Reflection of life* than men, less pronounced also for *Spirituality* and *Relationships*, but not for *Digital media usage* or *Perceived* *Restrictions* (Table 3). The higher the age is, the more intense the addressed changes were perceived, and the less severe the restrictions.

Persons who relied on their faith as a strategy to cope had significantly higher perceptions of change in all addressed fields, and lower perceptions of *Restrictions* (Table 3). Similarly, male and female (catholic) religious scored significantly stronger on *Nature/Silence/Contemplation*, *Spirituality*, and *Reflection on life *and had lower *Perceived* *Restrictions*, while they did not differ with respect to *Relations* and *Digital media usage*. When participants had low wellbeing, all perceived changes scores significantly lower compared to persons with moderate or high wellbeing, while the usage of digital media was similar (Table 3).

### Perceptions of change and their correlation with perceived burden, life satisfaction, spirituality and health behaviors

The factors were moderately to strongly intercorrelated, particularly *Nature*/*Silence*/*Contemplation* and *Relationships*, while *Digital media usage* was best related with *Spirituality,* and weakly only with the other perceptions (Table 4). *Perceived* *Restrictions* were marginally only related with the perceived changes.

**Table 4:**
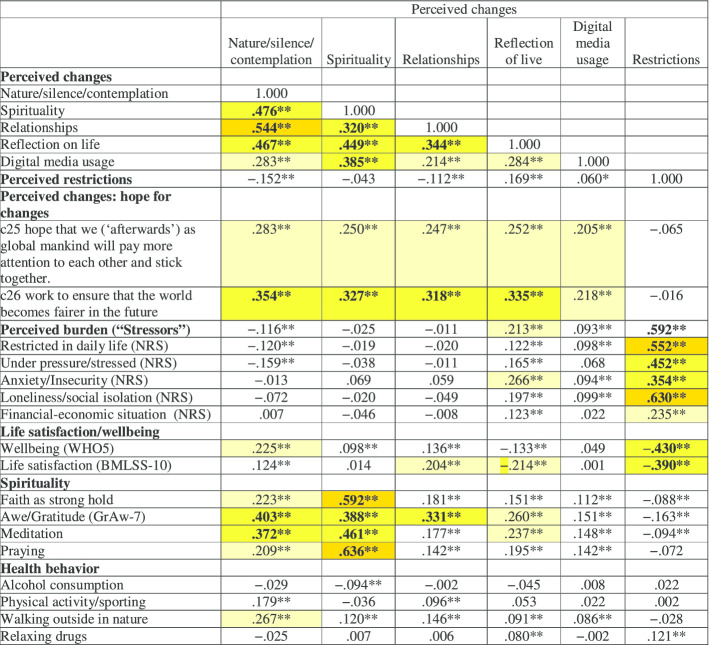
Correlations between perceived changes and external variables

**p < 0.001 (Spearman rho); moderate to strong correlations were highlighted (bold)

Burden and stress related indicators were moderately to strongly correlated with *Perceived Restrictions*, and either not at all or marginally only with the perceived changes (Table 4).

Participants’ wellbeing (WHO-5) was weakly associated with *Nature*/*Silence*/*Contemplation.* Their general life satisfaction (BMLSS-10) was weakly positively related with *Relationships* and negatively with *Reflection of life*. In contrast, *Perceived Restrictions* were moderately related with reduced wellbeing and life satisfaction (Table 4).

Faith as a strong hold was strongly related to *Spirituality*, while Awe/Gratitude as a perceptive form of (secular) spirituality was moderately related to *Nature*/*Silence*/*Contemplation*, *Spirituality* and *Relationship,* and marginally only with *Digital media usage* or low *Perceived Restrictions.*

Participants’ frequency of health behavior, particularly usage of relaxing drugs, alcohol consumptions and physical activity/sporting were not relevantly associated with perceived changes. However, walking outside in nature was weakly correlated with *Nature*/*Silence*/*Contemplation* (which is sound from a theoretical point of view). Frequency of meditation was moderately associated with *Nature*/*Silence*/*Contemplation* and *Spirituality,* while frequency of praying was strongly related with *Spirituality.*

### Predictors of perceived changes

Because several variables significantly related to the perceived changes during the Corona pandemic, stepwise regression analyses were performed to identify which of these variables would best predict these perceived changes (Table 5). Religious brothers and sisters have significantly higher Faith as a strong hold (85% vs 47%; *p* < 0.0001, Chi^2^) and significantly higher awe/gratitude scores (73.1 ± 15.4 vs 66.0 ± 18.1; *p* < 0.0001, Mann–Whitney-U test) than the other participants. Awe/Gratitude seems to be a more general indicator of secular spirituality which is moderately related to frequency of meditation (r = 0.44) and praying (r = 0.32), and to Faith as a strong hold (r = 0.35). Thus, we included the burden and wellbeing related variables, indicators of spirituality (Faith as strong hold, awe/gratitude, and also frequency of meditation and praying), walking outside in nature as a relevant behavior during the lock down restrictions, and also gender, age and living in a monastery as independent variables.

**Table 5:** Predictors of perceived changes (stepwise regression analyses)

		Beta	T	*p*
Dependent variable: Nature/Silence/Contemplation Model 8: F = 44.5, *p* < .0001; R^2^ = .24				
8	(constant)		6.429	< .0001
	Awe/Gratitude (GrAw-7)	.191	5.960	< .0001
	Meditation	.178	5.431	< .0001
	Walking outside in nature	.140	5.126	< .0001
	Wellbeing (WHO-5)	.119	4.248	< .0001
	Faith as hold in difficult times	.080	2.703	.007
	Gender (men)	− .072	− 2.653	.008
	Age groups	.076	2.672	.008
	Living in a monastery	− .063	− 2.190	.029
Dependent variable: Spirituality Model 7: F = 196.9, *p* < .0001; R^2^ = .55				
7	(constant)		2.158	.031
	Praying	.348	11.616	< .0001
	Faith as hold in difficult times	.280	9.630	< .0001
	Meditation	.170	6.827	< .0001
	Awe/Gratitude (GrAw-7)	.109	4.586	< .0001
	Age groups	.097	4.391	< .0001
	Live satisfaction (BMLSS-10)	− .078	− 3.744	< .0001
	Living in a monastery	− .056	− 2.519	.012
Dependent variable: Relationships Model 5: F = 38.5, *p* < .0001; R^2^ = .15				
5	(constant)		6.822	< .0001
	Awe/Gratitude (GrAw-7)	.235	7.599	< .0001
	Life satisfaction (BMLSS-10)	.193	5.794	< .0001
	Perceived burden (“Stressors”)	.107	3.275	.001
	Walking outside in nature	.090	3.142	.002
	Faith as hold in difficult times	.088	3.009	.003
Dependent variable: Reflections of life Model 5: F = 57.2, *p* < .0001; R^2^ = .20				
5	(constant)		5.454	< .0001
	Awe/Gratitude (GrAw-7)	.266	9.101	< .0001
	Life satisfaction (BMLSS-10)	− .199	− 6.189	< .0001
	Age groups	.141	4.847	< .0001
	Perceived burden (“Stressors”)	.187	5.888	< .0001
	Praying	.092	3.137	.002
Dependent variable: Digital media usage Model 4: F = 13.6, *p* < .0001; R^2^ = .05				
4	(constant)		8.173	< .0001
	Awe/Gratitude (GrAw-7)	.070	2.180	.029
	Perceived burden (“Stressors”)	.185	5.062	< .0001
	Wellbeing (WHO-5)	.136	3.583	< .0001
	Praying	.108	3.500	< .0001
Dependent variable: Perceived Restrictions Model 4: F = 173.0, *p* < .0001; R^2^ = .38				
4	(constant)		7.539	< .0001
	Perceived burden (“Stressors”)	.536	18.216	< .0001
	Awe/Gratitude (GrAw-7)	− .072	− 2.876	.004
	Wellbeing (WHO-5)	− .103	− 3.359	.001
	Gender (men)	.057	2.371	.018

*Nature*/*Silence*/*Contemplation* was predicted by eight variables, explaining altogether 24% of variance. The best predictor was awe/gratitude (which alone explains 15% of variance), followed by meditation, walking outside in nature and wellbeing. Age, gender, faith as a strong hold or living in a monastery were less relevant predictors.

*Spirituality* was predicted by seven variables, which explain 55% of variance. Best predictor was praying, which would explain 43% of variance, and further faith as hold in difficult times (which adds 6% of explained variance), meditation and awe/gratitude age (which together would add further 4% of variance). Life satisfaction, age and living in a monastery were less relevant predictors in this model.

*Relationships* were predicted by five variables, which explain only 15% of variance, best awe/gratitude, which alone would explain 10% of variance, and further by life satisfaction, and perceived burden (“stressors”), while walking in nature and faith as a strong hold were less relevant predictors.

*Reflections of live* were predicted by five variables, which explain 20% of variance, best again by awe/gratitude (which would explain 7% of variance), and further by low life satisfaction (which adds further 8% of explained variance), and by higher age and by perceived burden (“stressors”) (which together would add 4% of explained variance). Praying was a less relevant predictors in this model.

*Usage of digital media* was predicted by four variables, albeit with neglectable predictive power (R^2^ = 0.05). These variables are thus not reliable to predict observed digital media usage changes.

*Perceived Restrictions* were predicted by four variables, which explain 38% of variance, best by perceived burden (“stressors”), which alone would explain 36% of variance, while the other three predictors were of minor relevance.

### Moderation analysis

**Fig. 1:**
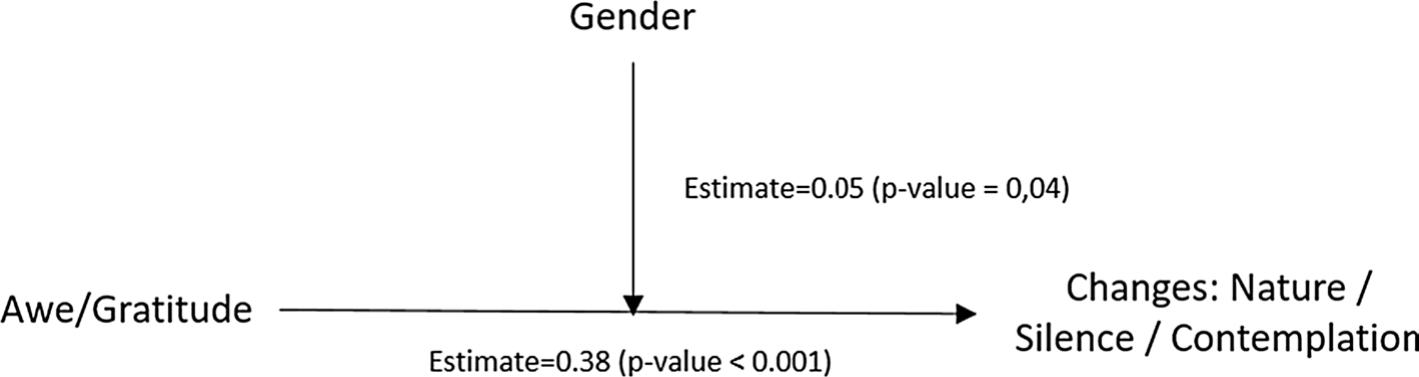
Moderation model for gender

Because persons living in a monastery and gender in general showed significant differences for awe/gratitude and for perceived changes, we tested whether both variables may moderate the relationship between awe/gratitude and perceived changes. Regarding *Changes: Nature, Silence*/*Contemplation* the strongest effect was found for gender as a moderator of awe/gratitude, albeit with weak effect (Fig. 1). For all other changes no significant moderation was detected (data not shown).

### Predictors of wellbeing

To address which of the perceived changes would contribute to participants’ wellbeing (as dependent variable), we performed a stepwise regression analysis with the perceived stressors, perceived changes, awe/gratitude as an indicator of spirituality, life satisfaction, and sociodemographic variables (gender, age and living in a monastery) as independent variables.

As shown in Table 6, 9 variables would explain participants’ wellbeing with good explanatory power (R^2^ = 0.51). Perceived burden (“stressors”) and life satisfaction were the best predictors (explaining 46% of variance), while awe/gratitude, *Nature*/*Silence*/*Contemplation*, and low *Reflection of life* had a further impact. Age, gender, *Digital media usage* and *Relations* were of minor relevance and can be neglected. Living in a monastery, perceived changes in *Spirituality*, and perceived *Restrictions* were not among the significant variables in this model.

**Table 6:** General predictors of wellbeing (stepwise regression analyses)

Dependent variable: Wellbeing (WHO-5) Model 9: F = 135.7, p < .0001; R^2^ = .51		Beta	T	*p*
9	(constant)		4.359	< .0001
	Perceived burden (“Stressors”)	− .355	− 13.971	< .0001
	Life satisfaction (BMLSS-10)	.329	12.596	< .0001
	Awe/Gratitude (GrAw-7)	.145	6.069	< .0001
	Age groups	.086	3.910	< .0001
	Changes: *Nature*/*Silence*/*Contemplation*	.143	4.927	< .0001
	Changes: *Reflections of life*	− .102	− 3.770	< .0001
	Gender (women)	.062	2.908	.004
	Changes: *Digital media usage*	.063	2.876	.004
	Changes: *Relations*	− .065	− 2.456	.014

Variables without a significant relevance in this model: living in a monastery, changes: *Spirituality*, and changes: *Restrictions*

## Discussion

We identified several topics of perceived changes during the Corona pandemic which were of relevance to the participants: (1) conscious experience of quiet times in life, mindful perceptions of nature and contemplative reflections (factor *Nature*/*Silence*/*Contemplation)*, (2) interest in spiritual issues, religious trust, and more intense praying/meditation to connect with the Sacred (factor *Spirituality*), (3) more intense and closer relations with partner, family and friends as resources of social support (factor *Relationships*), (4) reflections about meaning in life and the lifetime one may have; these were, however, associated with the perception of loneliness (factor *Reflection on life*), (5) usage of digital media to stay connected with others and to be inspired by specific website content (factor *Usage of Digital Media*). Further, the topics of isolation and loss were of relevance, but not as an intrinsic part of the positive *Perceptions of Change Questionnaire* (which was found to have good internal consistency coefficients and a plausible factorial structure). Strongest changes were perceived for *Relationships* (particularly relationships in which one can feel safe and at home, and more intensive perceptions of relations with partner/family) and for *Nature*/*Silence*/*Contemplation* (particularly paying attention to what is really important in life, and perceiving nature more intensively)*,* while *Restrictions* were perceived to a much lower extend. Interestingly, the positive changes were perceived significantly stronger by older persons and by those who relied on their faith as a resource (among them several religious brothers and sisters), while they perceived restrictions due to the pandemic less intensive. Older and retired persons may have more economic stability than younger persons who may fear for their workplace and financial-economic insecurity [14]. However, financial-economic insecurity was not a big issue in the investigated persons.

The perceived changes can be interpreted as ways to adapt with the outcomes of the COVID-19 pandemic restrictions. These reactions might not necessarily result in higher wellbeing, because several were still dealing with these restrictions and may feel stressed. Perceived changes of attitudes and behaviors are rather a form of a reappraisal strategy (in terms of coping) to ‘make the best’ of a bad situation and thus to downregulate negative affect and arousal [24]. Interestingly, reappraisal seems to involve the lateral temporal cortex which is related to semantic and perceptual representation rather than emotional control [24]. The negative effects of the Corona pandemic restrictions might be cognitively interpreted as an opportunity to make new experiences which otherwise would not be ‘learned’, and thus as a chance for personally ‘growth’. The pandemic-related ‘transformation’ or ‘growth’ (see [8–11]), however, was marginally too weakly only related to wellbeing or life satisfaction. More relevant were the associations of perceived changes with the perception of awe and subsequent feelings of gratitude. This variable was among the best predictors of persons’ perceived changes during the lockdown. This perceptive aspect of spirituality is not restricted to religious persons but is perceived also by a-religious persons [18]. It can be seen as experience of “mindfulness towards the present moment” [19] and is also a “life orientation towards noticing and appreciating the positive in life” [25]. In this study, *Nature*/*Silence*/*Contemplation* was related best to awe/gratitude, which was its best predictor in the regression model. It is in fact among the relevant predictors of all positive changes. This means, time-out breaks might be needed to perceive things differently and to become more aware of all those aspects in life which were taken so-far for granted, as inherently ‘available’ at any time. However, the pandemic restrictions changed these ‘automatisms’ and one is now focused more on the uniqueness of specific situations, relations, and experiences of nature. In fact, the Corona lockdown coincided with the spring season, when the blooming of nature could be observed much more intensely, and thus perceiving “nature more intensively” and going “outdoors much more often” is comprehensible.

An important observation was that the intention to start working for a fairer future world (as a reaction towards the pandemic) was moderately related to the four main perceptions of change (and weakly only for *Digital media usage*, and not at all for perceived *Restrictions*). This means, the processes of reflection not only changed general perceptions, but also raised hope in terms of a better world in the future once the Corona pandemic will be over. This coincidences with a hope that people will pay more attention to each other and stick together, as both are moderately related (r = 0.41). This hope for a collaborative humankind was similarly related to the perceived changes, albeit weaker. Both items were primary an integral part of PCQ’s factorial structure, but were finally not used due to a weak internal consistency of this 2-item factor.

Interestingly, several changes were perceived stronger by male and female religious, living in monastic structures with their explicit contemplative lifestyle compared to other persons. Because of their lifestyle they might be sensitized (or trained) to perceive the Sacred in their life and to reflect on what is essential. One might argue that their wellbeing is higher because of their lifestyle, and this is true. Religious brothers’ and sisters’ wellbeing (16.6 ± 4.6 vs 14.9 ± 5.2, *p* < 0.0001; Mann–Whitney-U test) and also their Awe/Gratitude scores (73.1 ± 15.4 vs 66.0 ± 18.1; *p* < 0.0001, Mann–Whitney-U test) was in fact significantly higher compared to other persons. This would argue that the grade of wellbeing might be of relevance, whether one may have faith to rely on or not. However, we were unable to verify “living in a monastery” as a moderator of the link between awe/gratitude and wellbeing or perceived changes (data not shown). However, those with low wellbeing (30%) perceived changes with respect to *Relationships* and *Nature*/*Silence*/*Contemplation*, but significantly lower compared to those with higher wellbeing, while they significantly reflected more on their life concerns compared to those with higher wellbeing. Further, *Spirituality* and *Relationships* scored significantly higher when persons felt well, compared to those with rather depressive scores of wellbeing. Also Persons with low wellbeing used digital media to connect with others and to overcome isolation, and this usage did not differ from others. This means, whether persons felt well or rather depressed during the pandemic has an influence what and how they perceive. When they have further faith as a resource to cope, all positive perceptions of change are stronger, and the negative restrictions were perceived less intensive. In the study of González-Sanguino et al. [14] spiritual wellbeing was found to be a protective factor against depression, anxiety and PTSD symptoms during the Corona pandemic, a finding which is not too surprising because most aspects of wellbeing are inversely related with reduced mental health indicators. Nevertheless, their data would underline the importance of this resource to cope during the Corona pandemic, too.

How do these perceived changes contribute to a person’s wellbeing during the pandemic? Regression analyses revealed that *Nature*/*Silence*/*Contemplation* would positively predict their wellbeing, while, however, *Reflection of life* was a negative predictor. This can be explained because it indicates that persons are more concerned about the meaning and purpose of their life and about the lifetime they have, and they perceive times of loneliness more intensely. These reflections have a negative connotation on the one hand, as they are related to anxiety and insecurity, and a positive connotation on the other hand, as these are positively related to awe/gratitude and meditation practices. Analyzing which of the so far tested variables would contribute to wellbeing revealed that first of all low perceived burden in terms of stressors and being satisfied with life in general were the best predictors among several others.

What are the consequences? Apart from general psychological support, perceiving nature, experiencing peaceful silence and wondering awe might be seen as resources to adapt during the pandemic. To support this, one might consider guided forest walks [26, 27] to encourage feelings of inner peace and stress-relief, also with the possibility of virtual walks for groups at risk. Further, mindfulness based meditation to relieve stress, reduce anxiety and depressive states, and to encourage conscious interactions with others could prove useful [28, 29], either as an individual practice at home and or in a group setting to avoid feelings of isolation and loneliness. Even web and app based mindfulness approaches seem to be effective [30, 31]. Important for most persons during the time of restrictions were concrete contacts with others on distance. Here, digital media resources proved their relevance, as this study has shown. Social media may facilitate visual communication on distance, while specific websites may provide content to inspire and stimulate others and thus allow the impression of participation. Others used such websites to attend digital services of worship, which may be a source of hold and hope for religious people. In fact, several relied on their faith as a resource to cope, while others do not have access to this source. A vital faith cannot be prescribed, but one could be made sensible for spiritual practices (i.e., meditation, prayer, distinct rituals) and train awareness of the uniqueness of the moment (referring to mindful awareness, conscious interactions and feelings of awe in specific situations). Therefore, retreats in monastic contexts could be an option to consolidate faith or to make new experience with a different lifestyle. Such contacts would also facilitate talks with pastoral professionals when phases of religious struggles [32, 33] or spiritual dryness [34, 35] may affect a person’s emotional and spiritual wellbeing. Further consequences which could be drawn from these findings remain to be discussed and tested in the next waves of the COVID-1 pandemic.

### Limitations

The study was performed as an online survey with a snowball sampling method and thus was not easily available for persons lacking internet access. We thus do not assume that the findings are representative for all German societies as the sampling strategy might have favored persons from academic contexts. The proportion of male and female catholic religious is probably higher than in a representative sample; however, it was our aim to have them in the sample as a contrasting group compared to persons with other live styles. Further, the cross-sectional design does nether allow any causal conclusions.

The number of the persons with COVID-19 infections in this sample (1.1%) seems to be a bit higher compared to the general German population in Germany within the respective time frame (0.2%).

Why the non-responders did not continue the online questionnaire is unclear. Comparing the non-responders with those who completed the survey did not show significant differences with respect to gender, age, lack of religious affiliations or COVID-19 infection testing.

Further, we have no knowledge about pre-existing mental health conditions of enrolled persons that may have impact the responses to the survey. We have decided to not ask for mental or physical diseases to increase willingness to participate.

## Conclusions

During the first phase of the COVID-19 pandemic people tried to find ways to adapt to the outcomes of the restrictions. The perceived changes of attitudes and behaviors can be interpreted in terms of a reappraisal strategy. They can be measured with the extended version of the Perceived Changes Questionnaire (PCQ) which was found to have good quality indices and plausible factor structure and is currently applied also in other cultural and religious contexts.

These observed perceptions of change contribute to persons’ wellbeing only to some extend and represent an independent quality of relevance in their life. Particularly perceived *Reflection of life* can be a hint of reduced life satisfaction, anxiety and loneliness and indicates need for special attention and support. It was crucial that most of these perceived changes were related to the ability to stop and perceive wondering awe with subsequent feelings of gratitude. These abilities could be fostered as they may provide a further resource to cope and find stability during difficult times. Now in October 2020, the second wave of the COVID-19 pandemic hits most countries with strong increases of infected persons. Whether the perceived changes of attitudes and behaviors really have an enduring impact that contributes to mental stability and posttraumatic growth remains to be shown during the next waves of the pandemic.

## Data Availability

According to the data protection regulations, the data set cannot be made publicly available. Data are however available from the authors upon reasonable request.
